# Unmasking *Balamuthia mandrillaris* Through PCR in a Case of Multifocal Brain Lesions

**DOI:** 10.1155/crdi/4411823

**Published:** 2026-05-29

**Authors:** Moamen Al Zoubi, Garry Jean-Louis, Kelly Sylvain, Cara Moll, Alison Schwartz VanEperen, Ammar Nasrallah, Amanda Carlson, Julie Duval, Norah Grady Letendre

**Affiliations:** ^1^ Department of Infectious Diseases, SSM Health St. Mary’s Hospital, Madison, Wisconsin, USA, ssmhealth.com; ^2^ Department of Pharmacy, SSM Health St. Mary’s Hospital, Madison, Wisconsin, USA, ssmhealth.com

**Keywords:** *Balamuthia mandrillaris*, brain abscess, free-living amoeba, granulomatous amoebic encephalitis, immunocompetent host, PCR diagnosis, rare CNS infection, ring-enhancing lesion

## Abstract

*Balamuthia mandrillaris* is a rare free‐living amoeba that causes granulomatous amoebic encephalitis (GAE), a frequently fatal central nervous system infection. Diagnosis is often delayed because of nonspecific clinical presentation and radiographic findings. We describe a 70‐year‐old man with recent soil exposure in rural Mexico who developed progressive neurologic decline and multifocal enhancing brain lesions initially concerning for malignancy. Extensive infectious, oncologic, and autoimmune evaluations were unrevealing. Brain biopsy demonstrated dense granulomatous inflammation without identifiable organisms on routine stains. Definitive diagnosis was established via 18S rRNA PCR testing of brain tissue and subsequently confirmed by the Centers for Disease Control and Prevention. A multidrug regimen including miltefosine and investigational nitroxoline was initiated but was complicated by renal and hepatic toxicity. Despite transient radiologic improvement, the patient experienced progressive disease and ultimately died. This case underscores the diagnostic difficulty of Balamuthia encephalitis and highlights the essential role of molecular diagnostics in identifying rare pathogens when conventional testing is inconclusive.

## 1. Background

*Balamuthia mandrillaris* is a rare, often fatal free‐living amoeba that causes granulomatous amoebic encephalitis (GAE). Early diagnosis and effective treatment remain challenging.

## 2. Case Presentation

A 70‐year‐old man, originally from Mexico and residing in the United States, presented with progressive neurological decline following recent travel. His past medical history included hypertension, Type 2 diabetes mellitus, and a prior left hip replacement. He had a remote history of coccidioidomycosis in 2007, diagnosed by biopsy of a cutaneous lesion and reportedly treated to resolution. In late 2024, he traveled to rural Mexico for four months to assist his brother with home repairs using traditional construction materials composed of dirt and donkey dung. He returned to the United States by bus on March 16, 2025. Family reported approximately 30 pounds of unintentional weight loss over a 4‐month period during his stay in rural Mexico. Three days later, he developed gastrointestinal symptoms, including nausea, vomiting, diarrhea, malaise, and a persistent headache.

**FIGURE 1:**
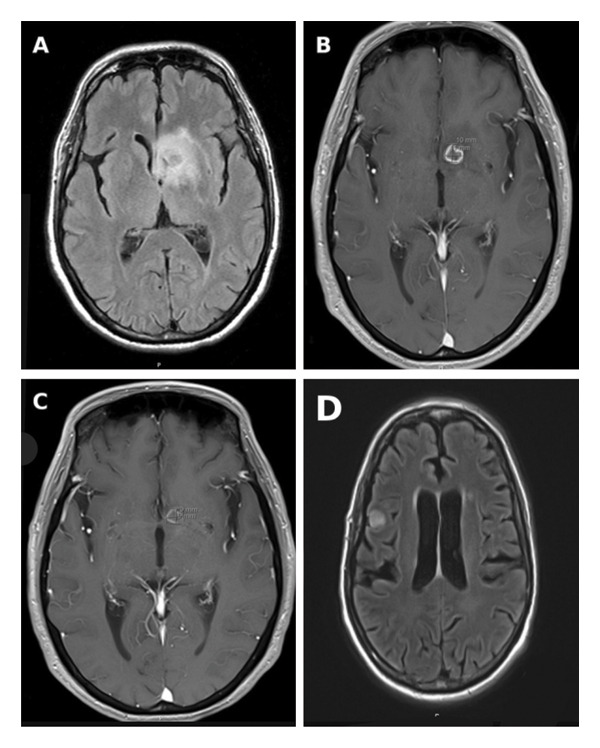
Initial MRI brain (A–D). (A) Axial FLAIR image demonstrating a hyperintense lesion centered in the left basal ganglia with surrounding vasogenic edema. (B) Axial postcontrast T1‐weighted image demonstrating rim enhancement of the basal ganglia lesion. (C) Axial postcontrast T1‐weighted image at a slightly inferior level demonstrating persistent enhancement of the lesion. (D) Axial postcontrast T1‐weighted image demonstrating a second rim‐enhancing lesion in the right frontal lobe, consistent with multifocal disease.

On March 29, he was hospitalized for symptomatic hyponatremia (Na 121 mmol/L), hypokalemia (K 2.6 mmol/L), and a chest X‐ray suggestive of right lower lobe pneumonia. He received ceftriaxone and azithromycin with clinical improvement and was discharged. However, on April 6, he was readmitted due to altered mental status characterized by lethargy and decreased responsiveness. Brain CT showed edema in the basal ganglia with mass effect, and MRI revealed multiple enhancing brain lesions, the largest measuring 1.6 × 1.6 × 1.4 cm in the left basal ganglia with surrounding edema and a 3‐mm midline shift (Figure 1(A)). He was transferred to a tertiary care center on April 7 for further evaluation.

An extensive workup, including chest CT, EEG, bronchoscopy with BAL, transbronchial lymph node biopsy, and cerebrospinal fluid analysis, was nondiagnostic. CSF studies showed lymphocytic pleocytosis, but bacterial, fungal, viral, and autoimmune panels were negative. Metagenomic sequencing of microbial cell‐fee DNA (Karius) returned negative. A PET scan on April 16 demonstrated FDG‐avid mediastinal and hilar lymphadenopathy. Brain biopsy on April 21 demonstrated dense granulomatous inflammation composed of lymphocytes, plasma cells, and histiocytes with areas of necrosis. No organisms were visualized on hematoxylin and eosin staining, Grocott methenamine silver stain, periodic acid–Schiff stain, or acid‐fast bacilli stain. No definitive amoebic trophozoites or cyst forms were identified on routine histopathologic evaluation. Additional workup identified *H. pylori* gastritis, a benign colonic polyp, and right‐sided panuveitis. A QuantiFERON‐TB Gold test was positive, but no evidence of active *tuberculosis* was found.

Amoeba PCR (18S RNA for *B. mandrillaris*) testing, performed on a brain biopsy, returned positive and was subsequently confirmed via PCR testing at the Centers for Disease Control and Prevention (CDC). On May 13, the patient was readmitted and a multidrug regimen was initiated, including sulfadiazine, flucytosine, voriconazole, azithromycin, miltefosine, and nitroxoline (obtained as an investigational drug under emergency IND via the CDC). The patient was closely monitored in the hospital for toxicity and tolerability. Within days, his renal function deteriorated, and he developed acute kidney injury attributed to flucytosine and sulfadiazine, ultimately requiring hemodialysis. A kidney biopsy revealed acute tubular injury superimposed on chronic changes. Sulfadiazine was subsequently resumed. Hepatotoxicity from voriconazole prompted a switch to posaconazole, which was later dose‐adjusted due to subtherapeutic levels. Repeat MRI on May 15 showed slight reduction in the left basal ganglia lesion with persistent perilesional edema (Figure 1(B)).

His clinical course was complicated by persistent nausea, anorexia, and functional decline, necessitating the initiation of enteral nutrition via a PEG tube placement on June 17. He experienced multiple falls, none of which resulted in intracranial hemorrhage. On June 5, MRI showed an interval decrease in lesion size and edema (Figure 1(C)). However, a subsequent MRI on July 3 revealed new findings concerning for disease progression, including enhancement of the third and fourth ventricles consistent with ventriculitis, a new right frontal lobe abscess, and multiple rim‐enhancing lesions suggestive of abscesses or infarcts involving the brainstem, cerebellum, and periventricular regions (Figure 1(D)).

Additional complications throughout hospitalization included anemia requiring transfusion and darbepoetin, and a right elbow effusion with associated myositis. MRI of the elbow showed edema of adjacent muscles and tendinopathy. Joint aspiration revealed neutrophilic fluid, but cultures remained negative. Low‐grade fevers occurred intermittently but were not clearly attributable to secondary infection. The patient’s condition continued to deteriorate despite maximal medical therapy. Goals of care discussions were held, and although there had been consideration of transitioning to comfort care, he developed acute respiratory distress on July 2. In accordance with prior discussions, care was not escalated. He passed away on July 3, 2025, from progressive *B. mandrillaris* GAE despite comprehensive, multidisciplinary treatment efforts.

## 3. Discussion

*B. mandrillaris* is a free‐living, pathogenic amoeba that causes GAE, a rare but almost universally fatal central nervous system infection. First isolated in 1986 from a mandrill that died of meningoencephalitis, *Balamuthia* has since been identified in over 200 human cases worldwide, with a mortality rate exceeding 90% [1, 2]. Transmission is believed to occur via inhalation of dust or through direct inoculation of contaminated soil into broken skin, with subsequent hematogenous dissemination to the CNS.

This case highlights multiple classical features of *Balamuthia* GAE: subacute progression, nonspecific initial symptoms, multifocal enhancing brain lesions, and exposure to potentially contaminated soil in a rural agricultural setting. Importantly, the diagnosis was delayed despite extensive infectious and oncologic evaluation, with definitive confirmation obtained only after PCR/CDC testing of a brain biopsy specimen. This underscores the challenge in diagnosing *Balamuthia* and the need to maintain a high index of suspicion in patients with compatible environmental exposures and imaging findings.

Histologically, *Balamuthia* produces necrotizing granulomatous inflammation with perivascular lymphoplasmacytic infiltrates, often mimicking vasculitis or malignancy. In this case, biopsy showed florid histiocytic and lymphoplasmacytic infiltration but no organisms on standard stains, consistent with prior reports [3].

Imaging typically shows ring‐enhancing lesions with surrounding vasogenic edema, commonly involving the basal ganglia, frontal lobes, and cerebellum. This patient’s MRI was notable for bilateral peripherally enhancing lesions with associated edema and mass effect—an imaging pattern seen in multiple published cases of *Balamuthia* [4].

Treatment remains highly challenging. The CDC currently recommends a combination of at least five drugs based on in vitro activity, case reports, and survivor experience. Common agents include miltefosine, flucytosine, triazole antifungals (voriconazole or posaconazole), sulfadiazine, macrolides such as azithromycin, and nitroxoline, an investigational urinary antiseptic with the recent in vitro evidence of efficacy. In this case, treatment was complicated by nephrotoxicity and hepatotoxicity, requiring regimen modification. Despite this, initial follow‐up imaging demonstrated radiologic improvement, prior to further deterioration.

There are very few documented survivors of *Balamuthia* GAE. One of the most cited is a 26‐year‐old woman treated with a six‐drug regimen including miltefosine [5]. The CDC provides guidance and access to investigational therapies and has compiled recommendations based on survivor regimens and laboratory testing [6]. Given the rarity of disease and lack of prospective trials, treatment remains empiric and toxicity‐prone.

## 4. Conclusion

This case underscores the pivotal role of direct tissue PCR in diagnosing rare causes of multifocal brain lesions when conventional tests and stains are inconclusive. Despite extensive negative infectious, oncologic, and histopathologic workup, molecular testing provided the definitive diagnosis of *B. mandrillaris*. The presence of enhancing brain lesions and granulomatous inflammation on biopsy should prompt consideration of free‐living amoebae, particularly in patients with environmental exposures. Timely PCR testing can be life‐saving and should be integrated earlier in the diagnostic algorithm for granulomatous CNS disease of unclear etiology.

## Author Contributions

All authors contributed to and approved the final manuscript.

## Funding

The authors have nothing to report.

## Ethics Statement

This case report was approved by the Institutional Review Board.

Unfortunately, the patient described in this report passed away during the course of the illness, and we were unable to obtain written consent.

## Conflicts of Interest

The authors declare no conflicts of interest.

## Data Availability

All relevant data are included within the manuscript.
